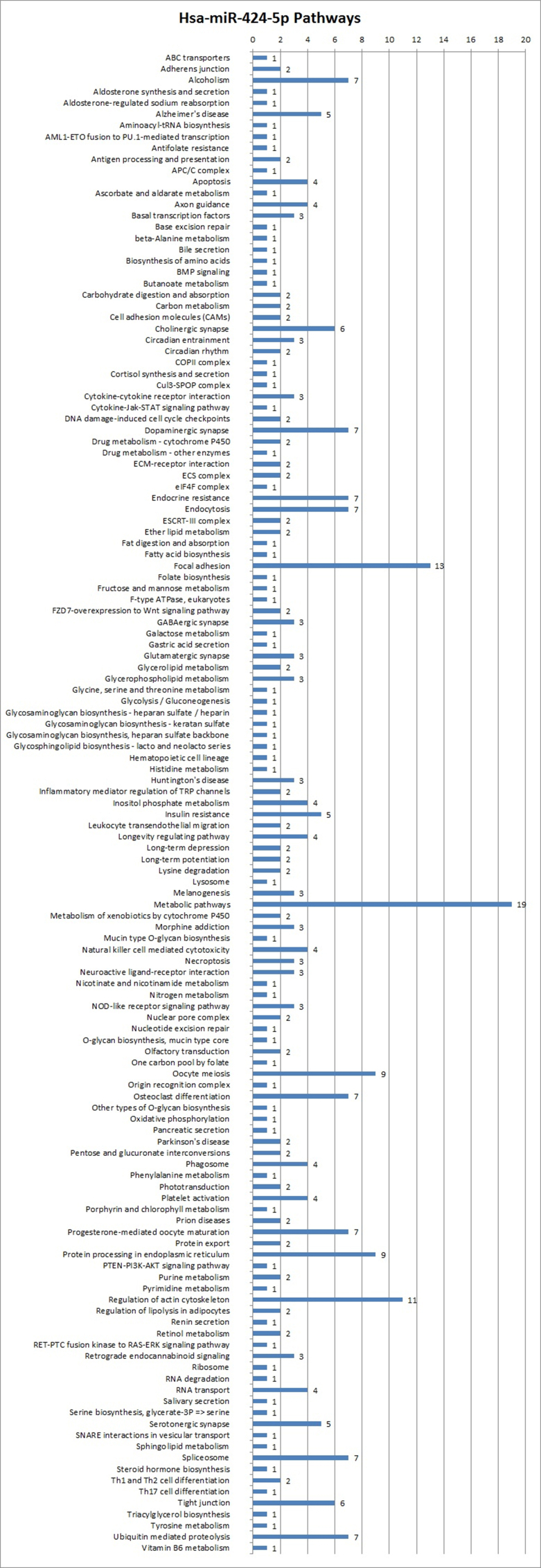# Identification of serum microRNA signatures associated with autism spectrum disorder as promising candidate biomarkers

**DOI:** 10.1016/j.heliyon.2021.e07462

**Published:** 2021-07-03

**Authors:** Tatyana Kichukova, Veselin Petrov, Nikolay Popov, Danail Minchev, Samir Naimov, Ivan Minkov, Tihomir Vachev

**Affiliations:** a Department of Plant Physiology and Molecular Biology, "Paisii Hilendarski" University of Plovdiv, 24 Tzar Assen Street, Plovdiv, Bulgaria; b Department of Plant Physiology, Biochemistry and Genetics, Agricultural University of Plovdiv, Bulgaria; c Psychiatric Ward for Active Treatment of Men, State Psychiatry Hospital Pazardzhik, Pazardzhik, Bulgaria; d Department of Medical Biology, Faculty of Medicine, Medical University-Plovdiv, 15-A Vassil Aprilov Blvd., Plovdiv, Bulgaria; e Division of Molecular and Regenerative Medicine, Research Institute at Medical University of 12 Plovdiv, 15A Vasil Aprilov Blvd, Plovdiv, 4000, Bulgaria; f Institute of Molecular Biology and Biotechnologies (IMBB), Plovdiv, Bulgaria

**Keywords:** ASD, Serum, miRNA, Biomarker, ROC analysis

## Abstract

**Background:**

MicroRNAs (miRNAs) are short non-coding RNA molecules with a well-recognized role in gene expression mostly at the post-transcriptional level. Recently, dysregulation of miRNAs and miRNA-mRNA interactions has been associated with CNS diseases, including numerous psychiatric disorders. Dynamic changes in the expression profiles of circulating miRNA are nowadays regarded as promising non-invasive biomarkers that may facilitate the accurate and timely diagnosis of complex conditions.

**Methods:**

In this study, we investigated the gene expression patterns of four miRNAs, which were previously reported to be dysregulated in pooled serum samples taken from Autism Spectrum Disorder (ASD) patients and typically developing children. The performance of a diagnostic model for ASD based on these four miRNAs was assessed by a receiver operating characteristic (ROC) curve analysis, which evaluates the diagnostic accuracy of the investigated miRNA biomarkers for ASD. Finally, to examine the potential modulation of CNS-related biological pathways, we carried out target identification and pathway analyses of the selected miRNAs.

**Results:**

Significant differential expression for all the four studied miRNAs: miR-500a-5p, miR-197-5p, miR-424-5p, and miR-664a-3p, was consistently measured in the samples from ASD patients. The ROC curve analysis demonstrated high sensitivity and specificity for miR-500a-5p, miR-197-5p, and miR-424-5p. With all miRNA expression data integrated into an additive ROC curve, the combination of miR-500a-5p and miR-197-5p provided the most powerful diagnostic model. On the other hand, the mRNA target mining showed that miR-424-5p and miR-500-5p regulate pools of target mRNA molecules which are enriched in a number of biological pathways associated with the development and differentiation of the nervous system.

**Conclusions:**

The steady expression patterns of miR-500a-5p, miR-197-5p, miR-424-5p, and miR-664a-3p in ASD children suggest that these miRNAs can be considered good candidates for non-invasive molecular biomarkers in the study of ASD patients. The highest diagnostic potential is manifested by miR-500a-5p and miR-197-5p, whose combined ROC curve demonstrates very strong predictive accuracy.

## Highlights

• MiRNAs are attractive candidates for ASD molecular biomarkers.
• MiR-500a-5p, miR-197-5p, miR-664a-3p and miR-424-5p show significant differential expression.
• MiR-424-5p and miR-500a-5p are the most dysregulated miRNAs in ASD patients.
• ROC analyses reveal miR-197-5p, miR-500a-5p and miR-424-5p as sensitive predictors.
• The combination of mir-500a-5p and mir-197-5p exhibits the highest diagnostic power.

## 1 Introduction

The Autism Spectrum Disorder (ASD) encompasses a heterogeneous continuum of neurodevelopmental disorders characterized by deficits in social exchange and communication, as well as fixed interests and stereotypic repetitive behaviors (American Psychiatric Association, 2013). Patients often exhibit enhanced sensitivity to sensory inputs, fixation on specific topics, and preference for familiar routines (Tsatsanis, 2005). In recent years, the prevalence of this condition has significantly increased, which may be attributed both to improved diagnostics and environmental factors like pollution (Meek et al., 2013). In the typical scenario, ASD is initially recognized in early childhood, with the average age of diagnosis being 4 years, and the symptoms are fully manifested during preadolescence (Hicks and Middleton, 2016).

There is strong evidence that ASD is a heritable disorder caused by thus far poorly understood gene-environment interactions (Yoo, 2015). One of the reasons is that its genetic component is highly diverse (Bourgeron, 2015). In most cases, a polygenic epistatic model with dozens of interacting genes is the most valid one (Persico and Napolioni, 2013). Numerous studies involving twins, siblings, families, and even populations, report hundreds of loci that may be linked to ASD and account for more than 50% of the cases (Geschwind and State, 2015). Intriguingly, the plethora of ASD-associated alleles represent only a few biological processes and pathways, including neuron activity, synaptic plasticity, calcium transportation, chromatin remodeling during neurogenesis, neuronal cell adhesion, and signaling pathways mediated by factors such as WNT, NOTCH, or SWI/SNF (Wu et al., 2016). Dysregulation of these processes probably leads to structural and functional deviations that ultimately affect the neuronal networks responsible for communication and social interactions (Ecker, 2017). However, despite the considerable effort, common ASD markers with high penetrance have still not been unambiguously identified (Vorstman et al., 2017).

In recent years, researchers have also been investigating a plausible alternative mechanism of ASD pathogenesis, which involves epigenetics. This kind of regulation coordinates gene expression without concurrent changes of the DNA sequence (Loke et al., 2015) and it has already been demonstrated to be able to influence entire gene networks (Mazzio and Soliman, 2012), including those that modulate neurogenesis and brain development (Im and Kenny, 2012).

MicroRNAs (miRNAs) are small (22–25 nucleotides in length), non-coding, regulatory RNA molecules that function by marking specific mRNAs for degradation or repressing translation (Rajgor and Hanley, 2016). MiRNAs possess remarkable operative potential since each of them can have multiple target molecules and thus drive large-scale fine-tuning of gene expression. It has been estimated that more than 60% of the human protein-coding genes can pair to miRNAs (Leclercq et al., 2017). Therefore, it is not surprising that miRNAs are involved in many diseases and are regarded as promising therapeutic agents (Hammond, 2015).

Remarkably, miRNAs are most abundant in the central nervous system (CNS), which produces around 70% of all miRNA species. Moreover, miRNA expression changes occur in the different phases of development as well as across the various brain regions (Ziats and Rennert, 2014). A growing body of evidence suggests that the dysregulation of these small RNA molecules is related to the pathogenesis of some major psychiatric disorders such as schizophrenia, bipolar disorder, major depressive disorders, Alzheimer's disease, and ASD (Geaghan and Cairns, 2015). In the case of ASD, it has been proposed that differentially upregulated miRNAs with high expression levels mostly repress genes associated with neuronal and synaptic dysfunction, while differentially downregulated miRNAs lead to abnormal activation of genes involved in inflammatory and compensatory processes (Wu et al., 2016).

Because of the social significance of ASD, considerable efforts are targeted at improving the promptness and correctness of its diagnosis. Unfortunately, current methods for ASD identification depend on behavioral observations and psychometric tools which cannot be fully carried out until 18–24 months of age and are not sufficiently specific (Hicks et al., 2016). Moreover, the intricate etiology of the condition is often additionally complicated by the occurrence of comorbid disorders, which intervenes with the precision of the diagnosis (Constantino and Charman, 2016). Therefore, novel and easily reproducible molecular tests, which complement the standard diagnostic procedures and/or provide reliable results before the ASD symptoms manifest themselves, are urgently needed.

**Figure 1:**
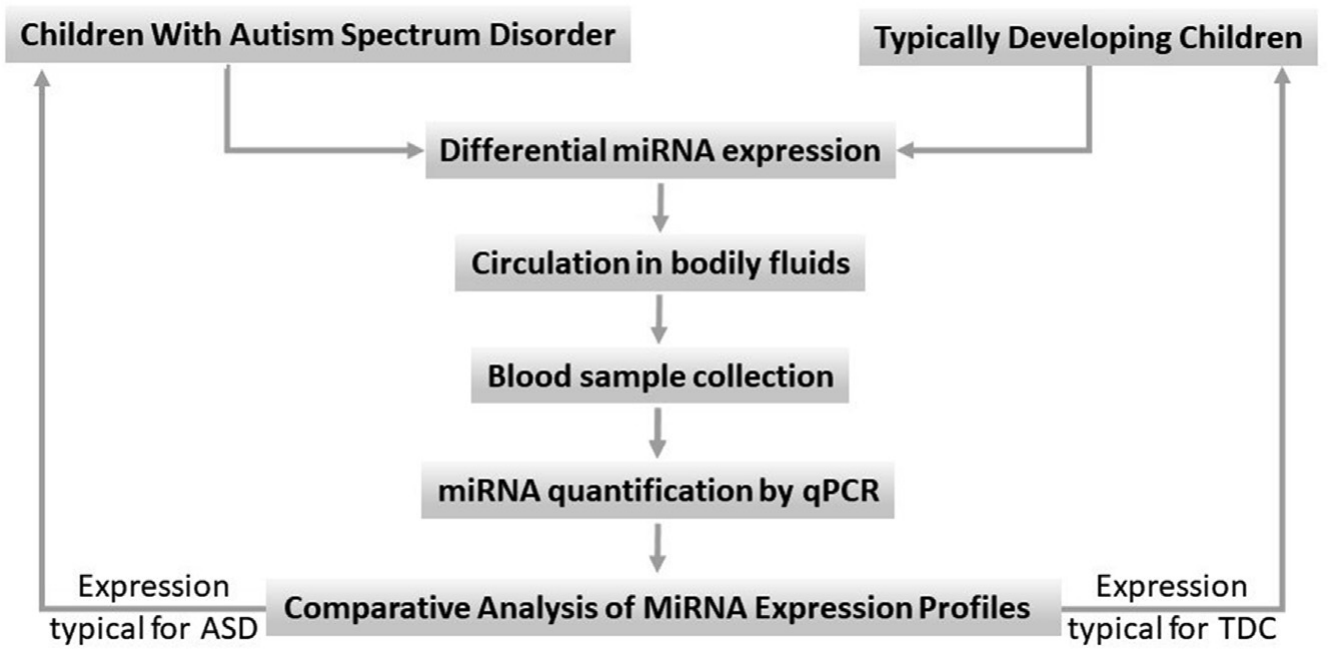
The rationale of the study, which is based on the following hypothesis: ASD pathological processes in CNS lead to differential expression of specific miRNAs. Some of these are freely circulating in bodily fluids, and thus - reliably measurable by quantitative RT-PCR. These marker circulating miRNAs may serve as an indicator for the development of ASD.

In our previous preliminary study, the small RNA-seq technology (Illumina HiSeq platform, BGI) was applied to analyze peripheral blood samples from control and ASD patients with the aim to identify potential circulating miRNAs that may serve as promising biomarkers for the ASD condition (unpublished data). Afterwards, an additional qRT-PCR profiling analysis was carried out on 42 miRNAs, which were the most highly differentially expressed among the studied groups, in pooled serum samples of 30 children diagnosed with ASD and 30 healthy controls. The results showed that miR-500a-5p, miR-197-5p, miR-424-5p, and miR-664a-3p were among the best candidates (Kichukova et al., 2017). Here we report a further validation of these findings through the screening of a larger cohort of subjects in which each sample was individually assessed. The predictive strength of the model, based on the above-mentioned miRNAs, for identification of ASD, is calculated by conducting a ROC analysis, and the putative role of these miRNAs in the pathogenesis of ASD is then discussed. The hypothesis of the present study is summarized in Figure 1

## 2 Methods

### 2.1 Ethics statement

The Ethics Committee of Plovdiv Medical University has approved the methodology of the study and the written Informed Consent Forms (ICFs). These ICFs were obtained by the parents of the probands after they were introduced to the major objectives of this study. The University Institutional Review Board has approved the collection and use of peripheral blood samples for the purposes of this work. All personal data were kept in the strictest confidence.

### 2.2 Patients and psychiatric assessment

A total of 66 children participated in this study. They were randomly selected from family practices. The cohort of probands was divided into two groups – one with ASD children (30 male and 8 female) and one with typically developing children (TDC; 22 male and 6 female). The first group included 38 patients, who had been diagnosed with ASD after interviews with the parents conducted by certified psychiatrists, and clinical examinations with the use of Autism Diagnostic Interview (ADI-R), Childhood Autism Rating Scale (CARS), and Gilliam Autism Rating Scale (GARS), following the diagnostic criteria described in the Diagnostic and Statistical Manual of Mental Disorders, Fifth Edition (DSM-V). The ADI-R (Autism Diagnostic Interview), once considered a gold standard assessment tool in identifying ASD, represents a structured interview conducted with the patients’ parents. The ASD diagnosis and its discrimination from other developmental delays, such as intellectual disabilities, are facilitated by the Childhood Autism Rating Scale (CARS) – a behavioral rating scale. The GARS (Gilliam Autism Rating Scale), on the other hand, is a norm-referenced screening instrument used for assessment of ASD in individuals aged 3–22 years and determination of the severity of the condition. GARS contains a section on developmental history and includes criteria for behavioral assessment in three categories: stereotyped behaviors, communication, and social interaction. Finally, the Diagnostic and Statistical Manual of Mental Disorders, Fifth Edition (DSM-5), updated in 2013, is the standard tool for psychiatric diagnoses used by mental health professionals in the USA, as well as for research purposes. The control group included 28 children who were chosen to match the age and sex of the ASD probands, and the absence of ASD symptoms was determined after a clinical examination and CARS assessment. The participants had not been subjected to any medication treatments before blood sampling. Children with known infectious, metabolic, or genetic conditions were also excluded from the trial.

### 2.3 Blood collection, serum processing, and RNA extraction

Blood (4 ml) was drawn by experienced physicians from a peripheral vein while the probands were in a fasting state (>3 h without food consumption). The material was collected in EDTA-containing tubes and stored at 4 °C before subsequent procedures. Thereafter, the samples were separated into serum and blood cells by centrifuging at 1600 g for 10 min at 4 °C. A volume of 500 μl serum was recentrifuged at 16 000 g for 10 min at 4 °C to remove any residual blood cells. The clear supernatant was transferred to RNase/DNase-free microfuge tubes in 200 μl aliquots and then stored at -80 °C until use. Total serum RNA (including miRNAs) was extracted from the 200 μl samples, following the addition of 5 μl *Caenorhabditis elegans* miR-39 (100 nM), a synthetic spike-in exogenous control for internal standardization (Schwarzenbach et al., 2015). The procedure for RNA extraction from the total serum was performed using PAXgene Blood miRNA kit (PreAnalytiX), according to the “Manual Purification of Total RNA, including miRNA” protocol recommended by the manufacturer, but with some minor modifications, such as initiation of the purification at step 4 and elution in 30 μl BR5 buffer.

### 2.4 Stem-loop real-time PCR quantification

**Supplementary Figure 1:**
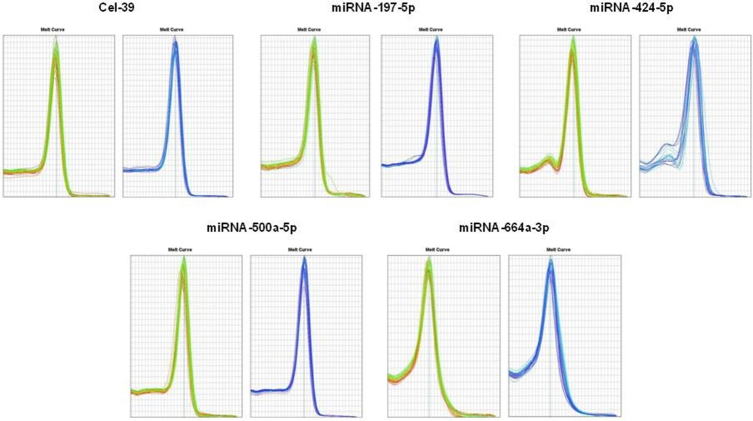


Maxima First Strand cDNA Synthesis Kit (Thermo Fisher Scientific) and miRNA-specific stem-loop primers were used for cDNA synthesis, following the manufacturer's instructions. The reaction mixtures for reverse transcription consisted of 8 μl RNA, 4 μl miRNA-specific cDNA synthesis primer mix (stem-loop and forward primers, 100 μm each), 4 μl Maxima Enzyme Mix, and 4 μl 5X Reaction Mix in a final volume of 20 μl. The miRNA-specific stem-loop (SL) and forward primers (For) used in this study are given in Table 1. Then, before carrying out the qRT-PCR quantification, 5 μl of each miRNA-specific cDNA were subjected to pre-amplification with peqGOLD Taq DNA Polymerase (VWR, Radnor, PA, USA) to increase the sensitivity of the assay. The qRT-PCR was performed using Maxima SYBR Green qPCR Master Mix (Тhermo Fisher Scientific) in an ABI 7500 system (Applied Biosystems). The samples were normalized against the spiked-in synthetic *C. elegans* cel-miR-39 miRNA control. All qRT-PCR reactions were carried out in duplicates. Each miRNA was measured in duplicates of each cDNA sample. Sample duplicates showing a difference of ≥ 0.2 between their Ct-values were excluded from the analysis. Finally, the relative quantification (RQ) values were calculated using the 2^−ΔΔCt^ methods (Livak and Schmittgen, 2001; Schmittgen et al., 2008). The specificity of amplification was confirmed by monitoring the dissociation curves of the amplicons (Melting curve analysis) (Supplementary Figure 1) and by agarose gel electrophoresis (data not shown).

**Table 1:** List of the primers used in this study.

MiRNAs	Primer sequence 5′- 3′
miR-197-5p SL	CTCAACTGGTGTCGTGGAGTCGGCAATTCAGTTGAGCCTCCCAC
miR-197-5p For	ACACTCCAGCTGGGCGGGTAGAGAGGGCAGT
miR-500а-5p SL	CTCAACTGGTGTCGTGGAGTCGGCAATTCAGTTGAGTCTCACCC
miR-500а-5p For	ACACTCCAGCTGGGTAATCCTTGCTACCTGG
miR-664а-3p SL	CTCAACTGGTGTCGTGGAGTCGGCAATTCAGTTGAGTGTAGGCT
miR-664а-3p For	ACACTCCAGCTGGGTATTCATTTATCCCCAG
miR-424-5p SL	CTCAACTGGTGTCGTGGAGTCGGCAATTCAGTTGAGTTCAAAAC
miR-424-5p For	ACACTCCAGCTGGGCAGCAGCAATTCATGT
Cel-miR-39 Spike-in	UCACCGGGUGUAAAUUA
Cel-miR-39 SL	CTCAACTGGTGTCGTGGAGTCGGCAATTCAGTTGAGCAAGCTGA
Cel-miR-39 For	ACACTCCAGCTGGGTCACCGGGTGTAAATC

SL – stem-loop; For – Forward; Rev – Reverse.

### 2.5 Statistical analyses

The statistical assessments were performed using version 20.0 of the SPSS package (SPSS Inc., Chicago, IL, USA). A nonparametric Mann-Whithney U test on the obtained delta Ct values was used to assess the significance of the miRNA expression differences between the two studied groups. A subsequent ROC (receiver operating characteristic) curve analysis was carried out using the MedCalc statistical software package to obtain specificity and sensitivity values of the analyzed circulating miRNA biomarkers.

Pathways prediction analysis of differentially expressed miRNAs in the serum: A detailed list of validated target genes of each studied miRNA was obtained from the miRWalk 2.0 database: http://zmf.umm.uni-heidelberg.de/apps/zmf/mirwalk2/. The miRWalk database supports a direct search functionality only for pathways involving putative but not validated target genes. Thus, we developed a custom tool that takes the initial lists of target genes as an input and then automatically explores the KEGG database: http://www.genome.jp/kegg/, for pathways enlisting the previously obtained validated miRNA targets. Subsequently, we plotted the total number of validated target genes with respect to the pathways in which they participate to construct the diagrams in Figure 5.

## 3 Results

### 3.1 Individual expression analysis of serum miRNAs in children with ASD

**Figure 2:**
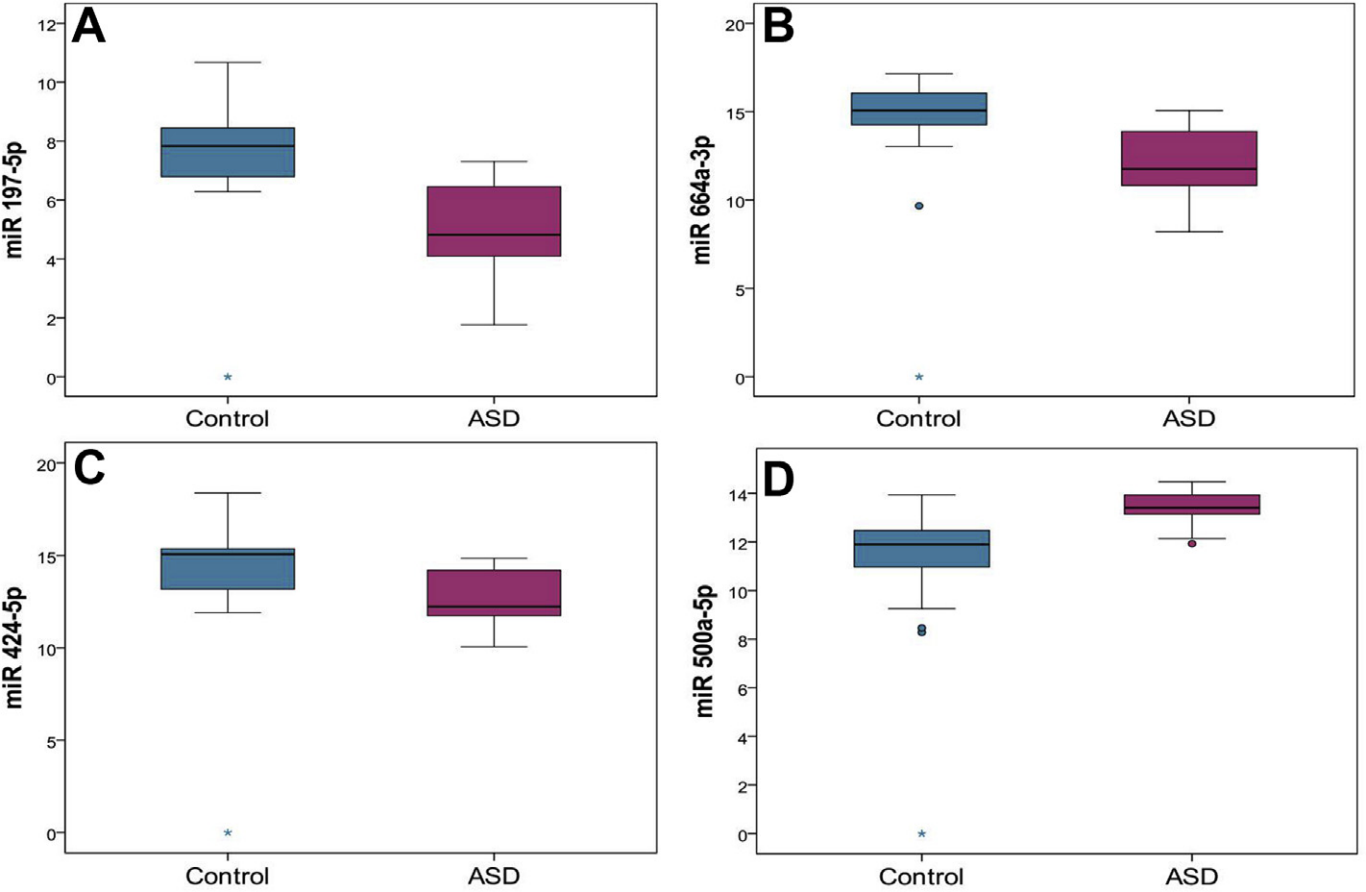
Differential expression of serum miRNAs in ASD patients: MiRNA specific stem-loop quantitative RT-PCR analysis of circulating miR-424-5p (Panel A), miR-500a-5p (Panel B), miR-197-5p (Panel C) and miR-664a-3p (Panel D) levels was conducted in case (n = 38) and control groups (n = 28), individually for each proband. Expression levels were normalized to spiked-in cel-miR-39 control. The y-axis on the box plot denotes the differences of the Ct cycle for each miRNA from the cel-miR-39 control, with a lower difference, i.e. higher expression, closer to the bottom of the plot. The line represents the median value. Significance was calculated by a nonparametric Mann-Whitney U test. Outliers are plotted as individual dots.

To evaluate the expression levels of specific serum-circulating miRNAs related to the development of ASD, an initial small RNA-seq experiment (data not published), comparing the target miRNA profiles of healthy and ASD-affected children, was followed by a qRT-PCR verification of the best candidates in pooled samples from the same groups (Kichukova et al., 2017). This pooled study included 30 children with ASD (24 male and 6 female) and 30 children with typical development (24 male and 6 female). In this study, a total of 42 miRNAs were assessed, of which 29 were found to be downregulated in ASD patients, 11 were upregulated, and 2 did not show expression changes in ASD children in comparison to healthy controls. However, pooling can significantly reduce the sensitivity of the approach and provides information only about the average value for the population sample, which masks the variation. Thus, a confirmation step including an evaluation of the expression in samples from individual patients is advisable. The four most dysregulated miRNAs, namely miR-500a-5p, miR-197-5p, miR-424-5p, and miR-664a-3p, were selected for such an analysis. Their expression was measured in 38 children diagnosed with ASD and 28 healthy controls with the use of stem-loop quantitative real-time PCR (qRT-PCR). Figure 2 presents box-plot diagrams of their Ct-value distributions relative to the endogenous controls.

As it can be seen in Figure 2, a tendency for ASD-related dysregulation is manifested for all investigated miRNAs. On average, the difference of the Ct-values for miR-197-5p from the exogenous cel-miR-39 used for normalization (delta Ct) was around 2.83 cycles lower in ASD samples than in the respective healthy controls (Figure 2A). At the same time, this parameter is 2.83 cycles lower for miR-664a-3p (Figure 2B); 2.16 cycles lower for miR-424-5p (Figure 2C), and 1.63 cycles higher for miR-500a-5p (Figure 2D). A subsequent analysis by a nonparametric Mann-Whitney U test demonstrated that the observed reduction of the miRNAs expression was statistically significant for all of the studied miRNAs, with the respective p-values being <0.001. There were small and statistically insignificant sex-related differences in the expression of the tested miRNA (data not shown). Due to both groups sharing roughly the same male to female ratio, such minor differences have no influence on the overall miRNA expression levels.

**Figure 3:**
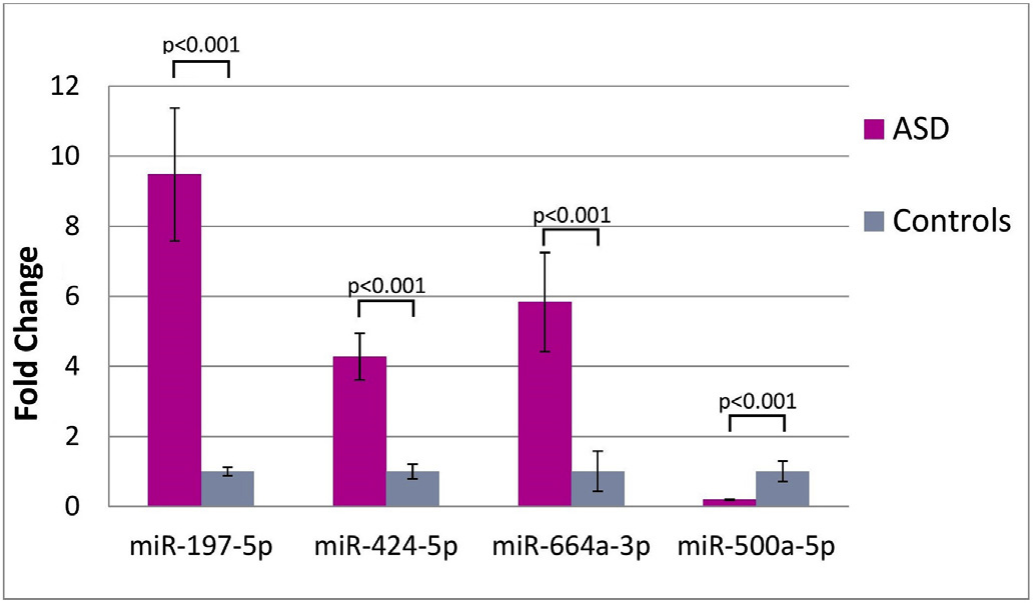
Fold change difference of the four investigated miRNAs between the ASD and control groups. Data are expressed as fold change of mean 2^−ΔΔCt^ for each miRNA after being normalized with spike-in cel-miR-39 control. Measurements are carried out in duplicates ±SD.

Two of the four target miRNAs, miRNA 197-5p and miRNA-664a-3p, exhibit the highest upregulation in ASD patients, 9.5-fold and 5.8-fold, respectively (Figure 3). In turn, miR-424-5p is around 4.3 times more expressed in children with ASD than in children with typical development, while miR-500a-5p is the only one that shows significant downregulation (more than fivefold). The observed expression pattern appears to be fairly stable across the different individuals, as indicated by the small variability of the data (the SD error bars). A wider range of the relative quantity distribution is only observed in the case of miR-197-5p.

### 3.2 Performance of an ASD prediction model using the four studied serum miRNAs

**Figure 4:**
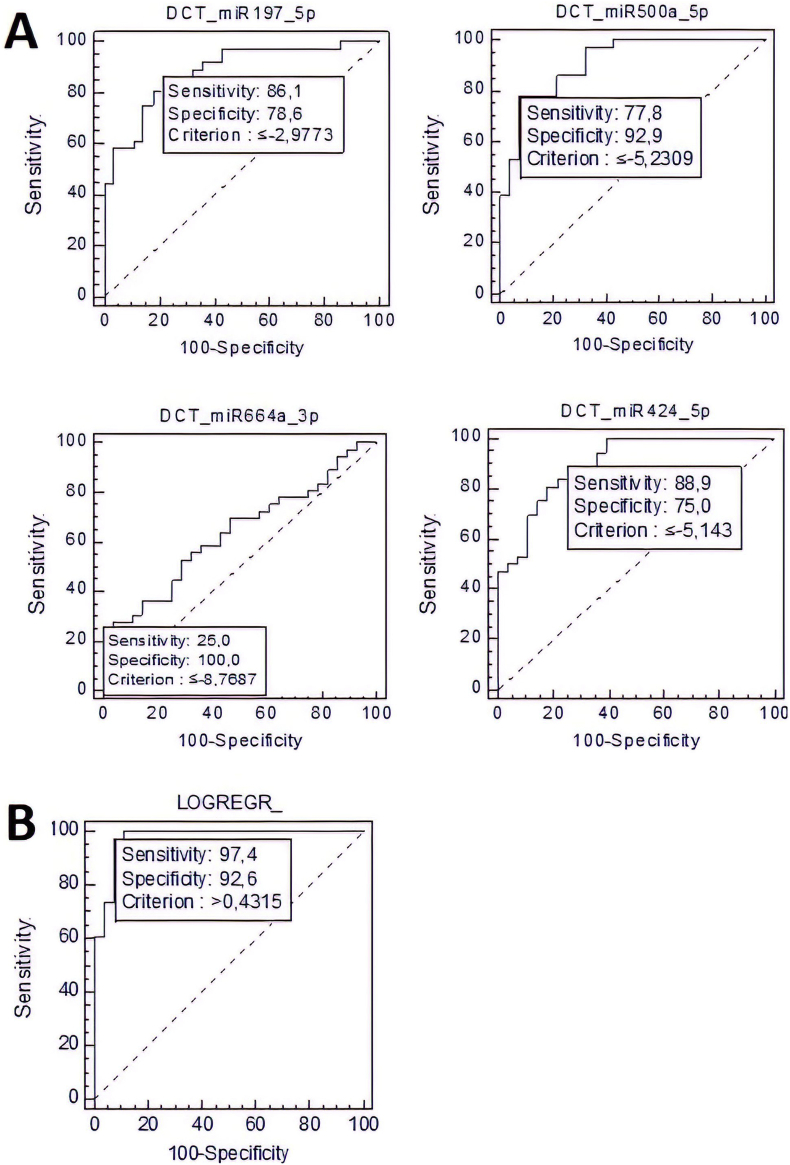
4A: ROC curve analyses of serum levels of miR-197-5p, miR-500a-5p, miR-664-3p and miR-424-5p used to distinguish ASD-patients (n = 38) from healthy controls (n = 28). 4B. Combined ROC-curve analysis. The best ROC curve is drawn with the data from miR-197-5p and miR-500a-5p but excludes miR-424-5p and miR-664-3p. The AUC of this variant was calculated to be 0,975, with a Standard Error of 0,0181 and a 95% Confidence interval ranging from 0,901 to 0,998. The diagonal lines on the plots represent the random classification accuracy (AUC 0.5).

To evaluate the strength of miR-424-5p, miR-500a-5p, miR-197-5p, and miR-664-3p as potential serum biomarkers for ASD, a Receiver Operating Characteristic (ROC) curve analysis was performed with the data from the qRT-PCR experiment. Subsequently, the area under the ROC curve (AUC), as well as the diagnostic sensitivity and specificity of each serum miRNA, were calculated and presented in Figure 4A. In addition, the prediction accuracy of a model based on all four miRNAs was evaluated by constructing a combined ROC curve (Figure 4B). As shown in Figure 4A, when calculated separately for each serum miRNA, the diagnostic sensitivities are as follows: 86.1% for miR-197-5p, 77.8% for miR-500a-5p, 25% for miR-664-3p, and 88.9% for miR-424-5p. In turn, the corresponding specificities are 78.6%, 92.9%, 100%, and 75%, respectively. The AUC parameters for miR-197-5p, miR-500a-5p, and miR-424-5p are 0.825, 0.796, and 0.756, respectively, while for miR-664-3p the AUC is below 0.7. According to these results, miR-197-5p, miR-500a-5p, and miR-424-5p are good ASD predictors, with sufficient sensitivities (i.e. true positive rates) of near or over 80%, and similarly favorable specificities (i.e. true negative rates) and AUC values (i.e. accuracy). MiR-664-3p is characterized by a worse diagnostic performance since it has a much lower probability of ASD detection – only 25% sensitivity and an AUC <0.7. However, the expression pattern of this miRNA in serum can be used to accurately rule out the possibility of ASD development in healthy individuals due to the extremely high (100%) specificity.

The expression data of the four serum miRNAs described in this study were simultaneously taken into account for the conduct of a combined ROC analysis (Figure 4B). Such integration for the miR-197-5p and miR-500a-5p markers increased the AUC value to 0.975. The sensitivity and specificity values of the new refined model were also impressive since both of them surpassed 90%. However, miR-424-5p and miR-664-3p expression did not improve the overall performance of the ROC model and thus they were excluded. Together, these findings indicate that when considered alone, three of the selected serum miRNAs, namely miR-197-5p, miR-500a-5p, and miR-424-5p, can discriminate between ASD cases and TDC with sufficiently high accuracy. The best diagnostic power for ASD is achieved by a model that is built with the combination of miR-197-5p and miR-500a-5p expression data.

### 3.3 Involvement of the studied serum miRNAs in relevant biological processes

**Figure 5:**
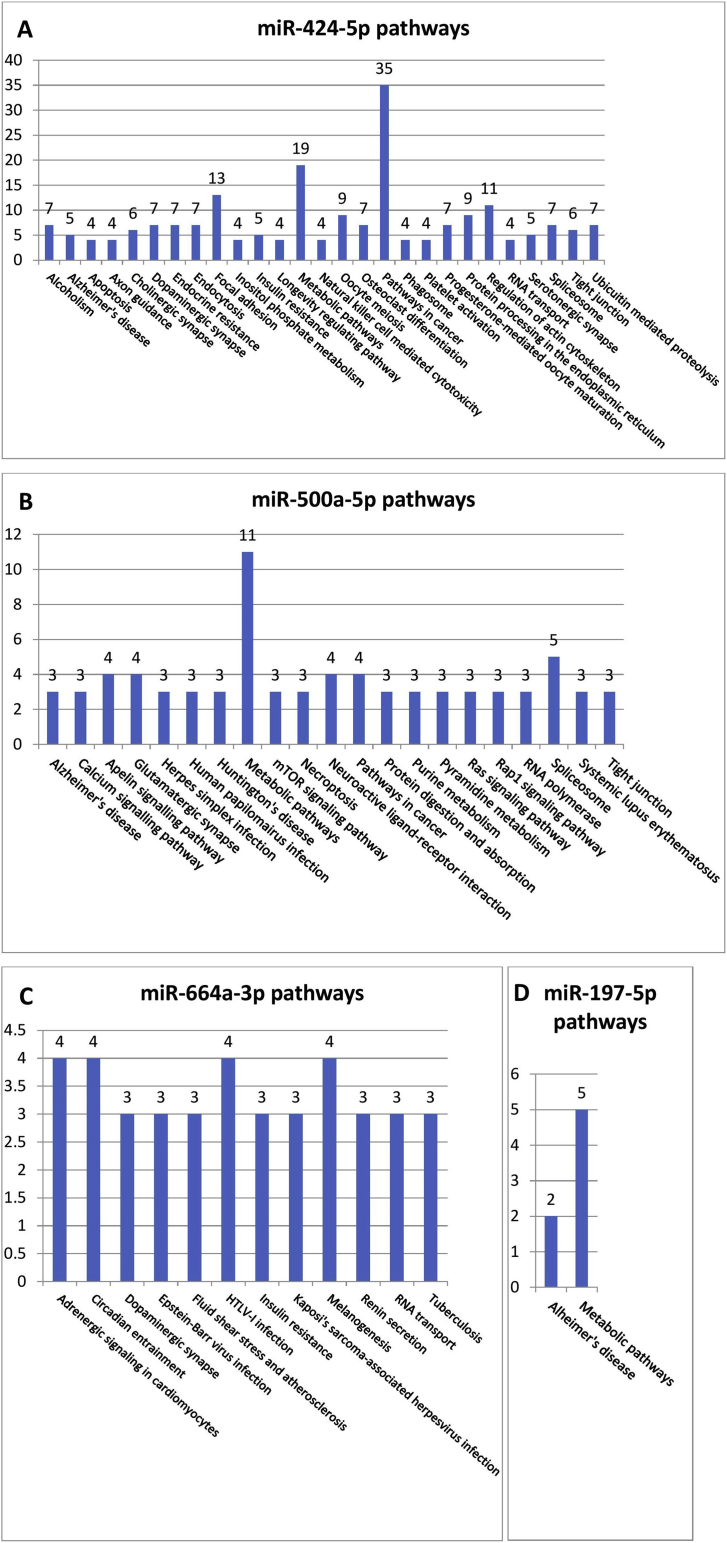
Enrichment of biological pathways in the mRNA target pools of the four studied miRNAs: A. miR-424-5p; B. miR-500a-5p; C. miR-664a-3p and D. miR-197-5p.

**Supplementary Figure 2:**
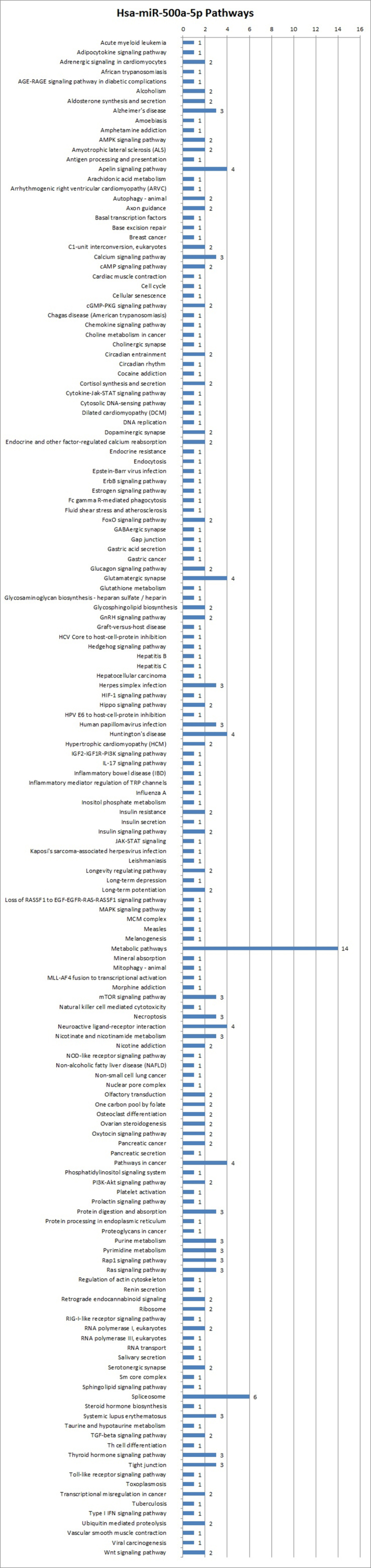


**Supplementary Figure 3:**
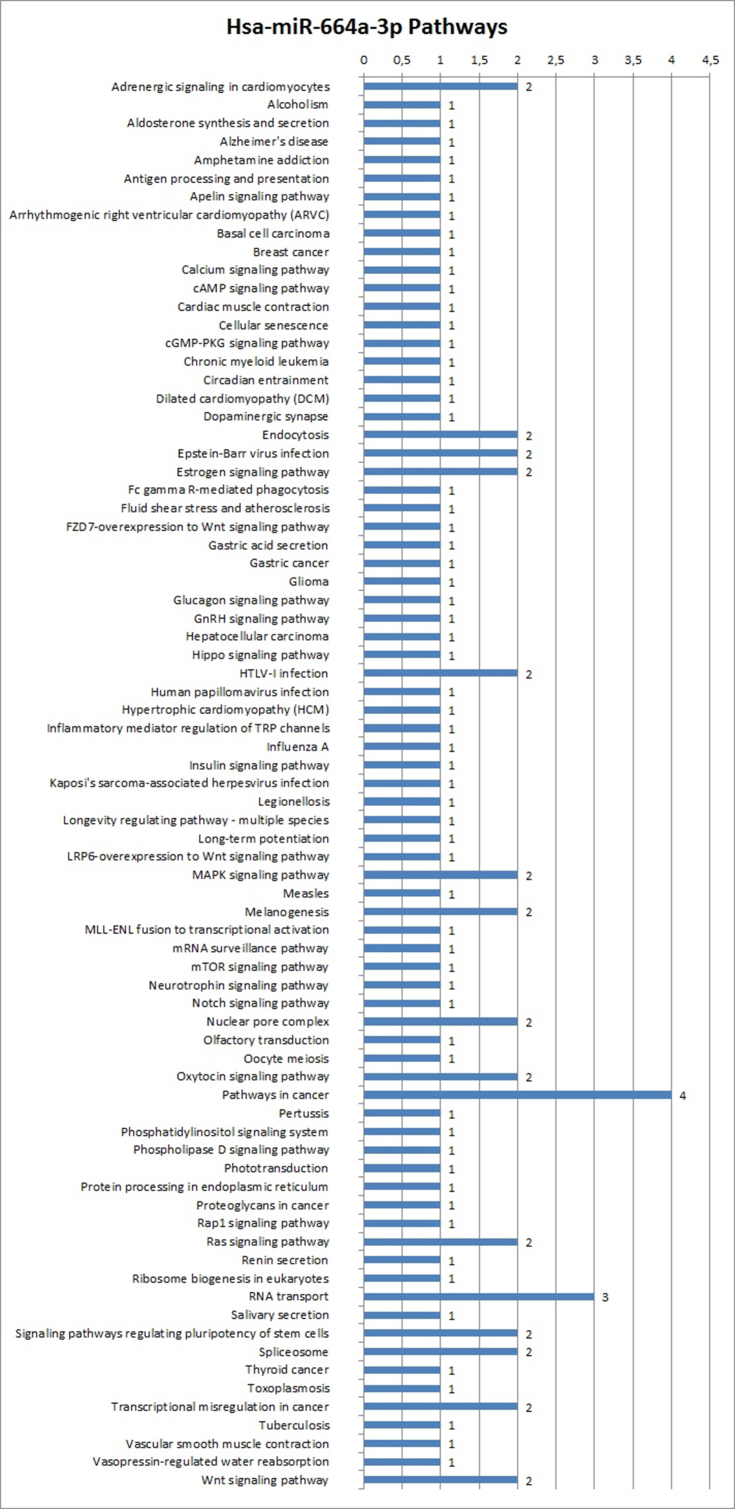


**Supplementary Figure 4:**
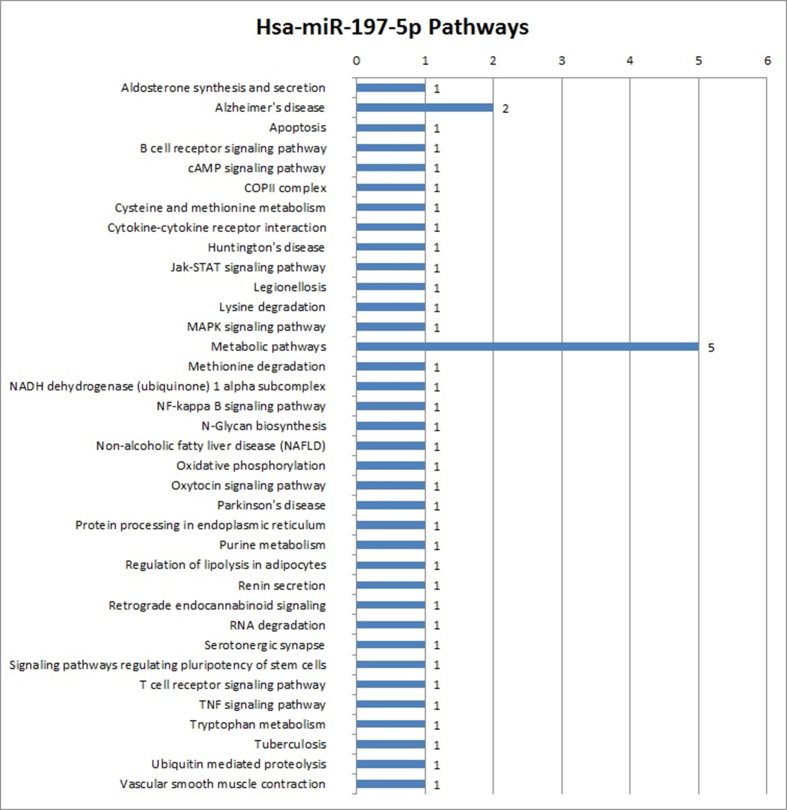


The mRNA targets of the studied differentially expressed miRNAs were obtained from the miRWalk database, as described in Materials and Methods, and their association with the relevant biological pathways is summarized in Figure 5. A more detailed list of biological pathways in which the validated miRNA target genes participate is given in Supplementary Figures 2, 3, 4, and 5. Querying the MirWalk database identified 671 unique validated target genes for all of the four differentially expressed miRNAs (35 of which are regulated by more than one miRNA). 287 of these validated targets were found to participate in 478 different biological pathways described in the KEGG database. Most of the obtained miRNA targets are related to common biological processes such as signal transduction, metabolism, and cancer diseases. However, 19 validated target genes are shown to participate in synaptic pathways (in cholinergic, dopaminergic, GABAergic, or glutamatergic synapses). Among them are four separate genes that encode G-protein subunits and thus play an important role in intracellular signal integration: *GNAL*, *GNAQ*, *GNB1*, and *GNG12*. Three additional genes (*GRIN2B*, *GABRG2*, and *GABARAPL1*) encode different subunits of receptors for neuroactive ligands or receptor-associated proteins. Finally, two other targets participate in glutamate reuptake: *SLC1A2*, *SLC1A1*.

Although miR-664a-3p has numerous validated target genes, their functional profile is dispersed in a large array of biological processes that are not directly related to the CNS or known key aspects of ASD behavior. Somewhat similar is the situation with miR-197-5p, with the distinction that the panel of known affected mRNAs is smaller. Moreover, miR-197-5p scores 2 hits in Alzheimer's disease category. In contrast, miR-424-5p and miR-500a-5p have a much more pronounced involvement in nerve tissue-specific pathways, including enrichment of target genes affecting different kinds of synapses, axon guidance, neuroactive ligand-receptor interactions, and some pathologies (Figure 5).

## 4 Discussion

In recent years, a growing body of evidence supports the hypothesis that dysregulation of the epigenetics-miRNA regulatory circuit may be a significant contributor to the initiation and progress of neurodevelopmental diseases (Abdolmaleky et al., 2015). Since miRNAs can be packaged in exosomes and microvesicles and thus be extruded outside cells, they can circulate in bodily fluids and be transported to distant tissues. This process allows the study of CNS-derived miRNA fingerprints through a simple collection of accessible material, for example, blood or even saliva (Grasso et al., 2014; Hicks et al., 2016). Expression profiling of extracellular miRNAs as putative biomarkers for various CNS disorders has already been reported in studies focused on epilepsy (An et al., 2016; De Matteis et al., 2018; Wang et al., 2015a), Parkinson's disease (Chen et al., 2018), bipolar mania (Rong et al., 2011), depressive disorders (Wang et al., 2015b), vascular dementia (Ragusa et al., 2016), attention-deficit/hyperactivity disorder (Wu et al., 2015), schizophrenia (Vachev et al., 2016; Wei et al., 2015), Tourette syndrome (Rizzo et al., 2015), ASD (Cirnigliaro et al., 2017; Kichukova et al., 2017; Mundalil Vasu et al., 2014), posttraumatic stress (Balakathiresan et al., 2014), and even internet gaming disorder (Lee et al., 2018). Particularly for ASD, dysregulated miRNAs have been detected in samples from different origin such as brain cortex and cerebellum (Ander et al., 2015; Schumann et al., 2017), peripheral blood (Huang et al., 2015), serum (Kichukova et al., 2017; Mundalil Vasu et al., 2014), saliva (Hicks et al., 2016), olfactory mucosal stem cells (Nguyen et al., 2016), monocytes (Jyonouchi et al., 2017), and lymphoblastoid cell lines (Sarachana et al., 2010).

In this study, we investigated the expression signature of four previously identified putative serum miRNA markers for ASD in individual probands. For all of them: miR-500a-5p, miR-197-5p, miR-424-5p, and miR-664a-3p, the ASD-related differential expression, earlier reported in pooled samples (Kichukova et al., 2017), was confirmed. Of note, the miR-424-5p and miR-500a-5p mediated regulation seems to have a significant impact on the CNS since a lot of their validated mRNA targets are implicated in processes occurring in the brain (Figure 5). In contrast, there is no apparent association between miR-197-5p or miR-664a-3p and CNS, which may partially be explained by the smaller number of affected mRNAs and the lack of enrichment in more specific KEGG pathways.

MiR-500a-5p has been shown to take part in biological processes in the CNS. During mouse embryogenesis, it is expressed in the brain, the spinal cord, and exhibits a peculiar asymmetric pattern of expression in embryonic structures associated with limb development/in the limb buds (Wheeler et al., 2006). In 2016, it was demonstrated that miR-500a-5p is a factor in neuropathic pain in rodent models with the use of a mechanism involving the downregulation of the glutamate decarboxylase isoform GAD67, which impairs the function of GABAergic synapses (Huang et al., 2016). Moreover, miR-500a-5p is among the miRNAs which are upregulated in the brains of patients with HIV-associated neurocognitive disorders (HAND) and contribute to the loss of peroxisomes and changes in their morphology by targeting peroxisome biogenesis factors (Xu et al., 2017). Here, we confirm an altered expression profile of miR-500a-5p in the serum of ASD patients, but unlike in HAND, in this case, the miRNA is downregulated. The affected mRNA targets, which are significant to the ASD pathology, are currently unknown.

Unlike the situation with miR-500a-5p, studies that specifically mention miR-197-5p are much scarcer in the literature. A search in PubMed with the keyword “miR-197-5p” returned only three results. Remarkably, two of those revealed the role of miR-197-5p in neurological disorders. The first one is devoted to late-onset Alzheimer's disease (LOAD) and presents data showing elevated expression of miR-197-5p in LOAD-affected brains (Roy and Mallick, 2017). The authors describe LOAD-related SNPs, one of which leads to a loss of the binding site of the miRNA in the 3′UTR of the transcription factor CP2. This, in turn, alleviates the miR-197-5p suppression of CP2, affecting biological networks that participate in the pathogenesis of LOAD. In the other work, miR-197-5p was shown to be significantly downregulated in plasma exosomes from mesial temporal lobe epilepsy with hippocampal sclerosis patients and was, therefore, one of the suggested potential therapeutic targets and biomarkers for the diagnosis of the condition (Yan et al., 2017). However, the parent miRNA of miR-197-5p, miR-197, is regarded as one of the major human oncomiRs, exerting a role either as a tumor promoter or suppressor, by targeting positive or negative oncogenes (Mavridis et al., 2015; Wang et al., 2016).

Comparably to the two previously described miRNA candidates, miR-424-5p has not been associated with ASD before. The apparent involvement of miR-424-5p in the control of cell division in different tissues might have a role in the development of ASD pathology as well. Recently, Wu et al. (Wu et al., 2016) hypothesized that the evolutionary role of some miRNAs in the primate brain is related to inhibition of excessive cell proliferation, a phenomenon observed in children with ASD. Therefore, the downregulation of miR-424-5p in ASD probands may reflect an insufficient capability to limit early postnatal brain overgrowth.

The most notable reference to miR-664a-3p concerning neuropsychiatric issues in the literature is a report dedicated to Alzheimer's disease and major depressive disorder (Mendes-Silva et al., 2016). The authors carried out a systematic review of the dysregulated miRNAs in the two conditions and found out that only seven were common for both of them, one of which was miR-664a-3p. In our experiment, however, despite the fact that the downregulation of that miRNA in ASD was statistically significant, it had the lowest alteration in the expression levels and the worst prognostic power (Figures 2, 3, and 4).

Numerous genes targeted by the four studied miRNAs were previously shown to be involved in biological pathways related to neurological diseases and psychiatric disorders. Three separate studies discovered an increase in the GNB1 protein expression in the prefrontal cortex of schizophrenia patients (Behan et al., 2009; Clark et al., 2006; Martins-de-Souza et al., 2009). Another study (Alfonso et al., 2006) described a reduction in the transcript levels for *GNAQ* in mice subjected to chronic stress. Mutations in the *GNAL*, a gene expressed prominently in the brain, are among the main causes of primary torsion dystonia and craniocervical dystonia (Fuchs et al., 2013; Kumar et al., 2014). Altered *GNAL* expression was also reported in schizophrenia (Mistry et al., 2013). One of the two miRNA target genes encoding glutamate transporters, *slc1a1*, has been extensively studied in the context of epilepsy, оbsessive-compulsive disorder (OCD), multiple sclerosis (MS), Alzheimer's disease, and schizophrenia (Bianchi et al., 2014). Significant overexpression of the SLC1A1 protein and its rodent homolog was found in postmortem brain samples from patients with epilepsy and rats with pilocarpine-induced epilepsy (Crino et al., 2002). In epileptic patients both up- and down-regulation of *SLC1A1* have been reported (Mathern et al., 1999; Proper et al., 2002; Bianchi et al., 2014; Rakhade and Loeb, 2008). Furthermore, increased expression of *SLC1A1* at both the mRNA and protein levels was demonstrated in schizophrenia patients (Bauer et al., 2008).

In conclusion, our findings demonstrate that four miRNAs: miR-500a-5p, miR-197-5p, miR-424-5p, and miR-664a-3p, are consistently dysregulated in the serum of children suffering from ASD and suggest that they could be used as easily available and measurable biomarkers for this disease. The diagnostic power of a molecular test based on miR-500a-5p and miR-197-5p will be the highest if these miRNAs are evaluated *en block*. Naturally, further supporting evidence from larger independent studies will be needed before clinical application. Moreover, additional research on these miRNAs may reveal details about the mechanism and exact consequences of their dysregulation in ASD patients, as well as whether their abnormal expression is involved in the pathology of the disorder or is the indirect result of other processes. Although the four miRNAs we found dysregulated in ASD are also differentially expressed in other neurological and psychiatric conditions, these miRNAs still provide notable specificity as potential biomarkers. The differential expression of these four miRNAs could be used in combination with other markers for ASD to verify the diagnosis. The reason behind such an assumption is that the other mentioned pathologies in which the studied miRNAs are also dysregulated rarely coincide with ASD. Moreover, the expression patterns of the same miRNAs in the other discussed conditions often contradict the ASD-specific expression we discovered. For instance, miR-500a-5p was found to be upregulated in HAND (Xu et al., 2017), but our data revealed its downregulation in ASD. MiR-197-5p is downregulated in epilepsy and hippocampal sclerosis (Yan et al., 2017), but appeared upregulated in this study. Finally, miR-664a-3p has been described as significantly upregulated (3.67-fold) in major depressive disorder and downregulated (1.5-fold) in Alzheimer's disease (Mendes-Silva et al., 2016), which sufficiently differs from the 5.8-fold upregulation observed in our experiment. Taken together these considerations support the potential role of the studied miRNAs as specific markers for ASD.

## Declarations

### Author contribution statement

Tatyana Kichukova, Veselin Petrov: Performed the experiments; Wrote the paper.

Nikolay Popov, Tihomir Vachev: Conceived and designed the experiments; Performed the experiments; Analyzed and interpreted the data; Wrote the paper.

Danail Minchev: Analyzed and interpreted the data; Wrote the paper.

Samir Naimov, Ivan Minkov: Conceived and designed the experiments; Contributed reagents, materials, analysis tools or data; Wrote the paper.

### Funding statement

This work was supported by the European Unions Horizon 2020 research and innovation program under grant agreement No 739582 (Project PlantaSYST).

### Data availability statement

Data included in article/supplementary material/referenced in article.

### Declaration of interests statement

The authors declare no conflict of interest.

### Additional information

No additional information is available for this paper.

## Figures and Tables

**Supplementary Figure 5:**